# Measuring the quality of clinical veterinary services for Cattle: an application of a role play experiment in rural Uganda

**DOI:** 10.1186/1756-0500-7-894

**Published:** 2014-12-10

**Authors:** John Ilukor, Regina Birner

**Affiliations:** Institute of Agricultural Economics and Social Sciences in the Tropics and Subtropics, University of Hohenheim, Stuttgart, Germany; CGIAR Standing Panel on Impact Assessment (SPIA), FAO, Rome, Italy

**Keywords:** Belief updating, Lemon market, Role play game

## Abstract

**Background:**

The dominance of veterinary paraprofessionals in the animal health markets has been linked to the decline in quality of veterinary services. This study uses a role play experiment to analyze how the interaction of farmers and service providers influences the quality and the demand for clinical services for cattle. The quality of clinical services was measured by scoring the accuracy of the service provider prescribing the appropriate drug for selected cattle diseases.

**Methods:**

The game was played in four rounds. Farmers were given “animal medical card” with the name of the disease written on it both in English and the local language in each round. Service providers were asked to write the clinical signs, and prescribe the drugs.

**Results:**

The results show that the ability to identify the signs of different diseases and the accuracy of prescriptions by veterinarians is not significantly different from that of paraprofessionals trained in veterinary science. However, the ability of service providers who are not trained in veterinary medicine to perform these tasks is significantly lower than that of service providers trained in veterinary science. The continued interaction between paraprofessionals and veterinarians gradually leads to an improvement in the ability of paraprofessionals trained in general agriculture and social sciences to perform these tasks. This was not the case for paraprofessionals with no formal training or education. Farmers do not easily change their beliefs about paraprofessionals, even if they receive information on their inability to diagnose diseases correctly and prescribe the correct drugs. Belief updating depends not only on the outcome of the previous round, but also on the gender of the farmer and the livestock production system.

**Conclusion:**

This paper argues that the slow pace in which farmers update their beliefs about paraprofessionals limits paraprofessionals’ willingness to learn or consult with veterinarians. However, the use of “animal health cards” (records of diagnoses and treatments) could induce paraprofessionals to provide services of better quality clinical services for cattle and enable farmers to measure the quality of these services.

## Background

This paper is concerned with measuring and assessing the quality of clinical veterinary services in developing countries, using Uganda as an example. The existence of veterinarians and paraprofessionals of varying skills and training can be a major problem in animal health markets [1]. Veterinarians have to compete with less qualified or unqualified service providers and professionals trained in general agriculture that may be providing less quality services compared with services of veterinarians [2]. Livestock farmers who have no skills or training in veterinary science are not able to measure the quality of services being offered by these service providers. The failure of a farmer to assess and measure the quality of the service creates motivation problems such that a farmer is not willing to pay a premium fee for the service because he cannot judge the quality of the service he or she receives. As a result, service providers that deliver high quality services are forced to accept low payment since they cannot convince the farmer that their services are of high quality [3]. Since service providers interact repeatedly with farmers, farmers’ failure to differentiate the quality of the service leads to high quality service providers being displaced or nudged off the market. Akerlof [4] describes this interface between quality heterogeneity and asymmetric information resulting to the disappearance of a market with quality goods and services as a market for “lemons”. As argued by Ly [3], a market for “lemon” has occurred in animal health markets in most developing countries, and the veterinary paraprofessionals have dominated the animal health market.

The dominance of the veterinary paraprofessionals in the provision of veterinary services, although useful in reducing costs and increasing access, has been linked to the decline in the quality of veterinary services [5, 6]. Improving relationship between veterinarians and paraprofessionals is seen as an important approach towards improving the quality of clinical veterinary services [7–9]. In addition, the economic literature on the provision of animal health services emphasizes that if farmers had information about the quality of service offered, they would be able to update their beliefs and more readily seek services of veterinarians who offer quality services. Belief updating (belief change) is the act of changing a previously held belief to take into account new information [10]. By seeking quality services, paraprofessionals or low quality service providers would strive to consult with veterinarians in order to maintain and build their reputation.

In this study, a role play game experiment was used to assess the influence of information on farmers’ beliefs about service providers and the quality of clinical services. In particular, the study aimed at answering the following questions: (1) Does the quality of services provided by paraprofessionals differ with that provided by veterinarians? (2) Does quality improve as paraprofessionals and veterinarians interact? (3) Do farmers update their beliefs about service providers? And (4) what factors influence farmer belief updating? The paper proceeds as follows: Section 2 covers materials and methods; Section 3 presents the results, and Section 4 discusses the findings and provides a conclusion.

## Methods

This study is part of the project aimed at analysing institutional arrangements for providing veterinary services in Uganda and Kenya. Ethical approval of prospective enrolment of humans to the study was obtained from the committees at directorates of the veterinary services at Ministry of Agriculture, Animal Industry and Fisheries in Uganda and the Ministry of Livestock in Kenya. The reference number for Kenya is RES/GEN/VOL.X11/63. The reference number for Uganda was not obtained, but verbal permission form directorate of animal resources was obtained. Consideration was also given to welfare of the subjects, and we are confident that the study did not pose any risk to the subjects since clinical trials were not involved. We also received permission from local governments and one staff in the veterinary department was assigned to work with us in each district. Consent of the subjects was also obtained.

### Design of the game

The experimental data used in this paper were collected from two different districts in Uganda (referred to here as A and B to ensure the anonymity of the participants). District A is located in the pastoral production system while District B is located in the intensive livestock production system. Subjects were recruited from each district. The subjects included the farmers, paraprofessionals and veterinarians. They were asked whether they agreed to participate in a role play game. Farmers were told they would be paid, and their pay-off would depend on the outcome of the transaction (treatment of a sick animal in the role play) and their ability to negotiate with service providers for a fee the providers would charge. The farmers were provided with an initial endowment of 6,000 Uganda Shillings (US$2), which was approximately three times the daily wage for unskilled labour in the study regions. If the outcome was positive, a farmer would be paid a reward covering the difference between the fee of the service provider and the initial endowment. A positive outcome was one where the animal was cured, which happened if the service providers identified the right drug for the disease of the animal under consideration. If the outcome was negative, the farmer received nothing. A negative outcome means that the animal died because the service provider was not able to identify the appropriate treatment. Service providers were informed that their earning would depend on their reputation with farmers, which determined the number of farmers who demanded their service, and the professional fee they charged. Service providers were allowed to refer and consult other service providers. The cost of transport and drugs was considered as dead weight costs and hence not included in the game.

A total of 51 farmers were recruited to participate in the experiment, 26 in the pastoral livestock production system (10 female and 16 male) and 25 in the intensive livestock production system (12 female and 13 male). In each production system, it was planned to recruit two veterinarians and five paraprofessionals to participate in the game. In District A (pastoral area), however, veterinarians are usually absent from their duty stations because there are few trained veterinarians from these areas, and professionals from non-pastoral ethnic groups are often reluctant to work in pastoral areas because of the harsh climate and poor infrastructure [11]. Therefore, two government animal health assistants with a two year diploma training in veterinary medicine were asked to act as veterinarians in the role play. Their performance in terms of disease diagnosis and drug prescription was later compared with that of veterinarians in District B, and it was found that there was no statistically significant difference in their scores. Therefore, it can be assumed that this replacement does not affect the results. The training level of the paraprofessionals differed between the districts. In the pastoral system, two of the paraprofessionals had diplomas in social science with three months of training in animal health, and the other three had either primary or no education, and they also had received three months of training in animal health. Three paraprofessionals in the intensive production system had certificates in general agriculture, and two had diplomas in general agriculture. In District B, there were no female service providers and in District A, two of paraprofessionals were women and the rest of the service providers were male. In the intensive system, three endemic livestock diseases that were identified and they are; East Coast Fever, Anaplasmosis, and Tryponamiasis. In the pastoral systems, extra two diseases were added, namely, Heart Water and Red Water.

The game proceeded as follows: Farmers were given a so-called “animal medical card” with the name of the disease written on it both in the local language (Pokot and Luganda) and in English. The animal medical cards were distributed to the farmers on a random basis. Farmers were asked to choose any service provider of his or her choice to treat a particular disease. The service provider chosen by the farmer had to list the signs associated with the disease (corresponding to performing a clinical diagnosis in real life) and prescribe the drugs. The service provider also had to agree on the costs of treatment with a farmer. The costs were broken down into the professional fee, cost of drugs and transport fee. All this information was written down on the animal medical card. Two of the paraprofessionals in the pastoral areas who could not read or write in English were assisted by hired university students with no veterinary training. The students were instructed to write only what the paraprofessionals told them to write. The cards were later handed back to the farmers who presented the cards to the researcher. The researcher would then assign the outcomes based on drug prescription. Outcomes were categorized as positive and negative. As indicated above, a positive outcome is one where the animal is cured (appropriate drug prescribed) while a negative outcome is one where the animal died (wrong drug is prescribed). The signs of the diseases and the treatment are presented in Table 1 below. It was designed by consulting the practicing veterinarians, the Merck Veterinary manual ^a^ and the OIE technical disease cards ^b^.

**Table 1:** **Clinical signs and drugs for specific animal diseases**

Disease	Clinical signs	Main drug(s)	Scores	Supplementary drugs	Scores
East Coast Fever	High temperature of about 40°C, swollen lymph nodes, increased breathing loss of appetite, nasal discharge, cough, white discharge in the eyes	Butarex, Parvexion, Clexion and Aflexion	8	multivitamins and oxy-tetracycline	2
Anaplasmosis	High temperature (41°C), severe constipation, loss of appetite, loss of body weight, increased breathing and dry mouth	Imisol	8	salts, multivitamins and oxy-tetracycline	2
Trypanosomiasis	High temperature, stunning hair, loss of body weight, lacrimation (crying), blood discharge from the ears or skin, mucus discharge and brown urine.	Suriname, Diminazene and Ethidium	9	oxy-tetracycline	1
Heart Water	Turning in circles, grinding of the teeth, sensitivity to touch, nasal discharge and high temperature	Oxy tetracycline	9	Multivitamins	1
Red Water	Reddish urine, high temperature, loss of appetite, laboured breathing and weight loss	Imisol, Diminazene and Berenil	9	Multivitamins	1

Source: Authors.

The game was played in four rounds, and at the beginning of each round, the farmers received a new medical card. At the end of each round, both farmers and service providers received information about the outcomes, and their pay-offs were awarded. After the game, the participants were invited to share their reflections, and finally, the meal was served.

### Analysis of data

To measure the quality of clinical cattle services and the effect of farmers’ decisions on the demand and quality of clinical veterinary services, the degree of accuracy in identifying the signs of the disease listed on the animal medical card and prescribing the appropriate treatment were used as indicators of quality of service provision. After every round, the participants were able to consult and share their results with others. The scores for every round were awarded as presented in Table 1. Each cardinal sign identified was awarded a score of one and the total score was transformed into percentage. In the case of drug prescription, scores were awarded based on the drugs prescribed by the service providers. As shown in Table 1 below, if a service provider prescribed one of the main drugs, he was given a score of 8 or 9. He also received 1 or 2 points for all supplementary drugs, depending on the disease. These scores were transformed into percentages and since eight points was the lowest score for prescribing the main drug, the pass mark could be set at 80%.

The data from the role play were entered into a database and analysed as follows: Scatter diagrams were used to analyse the quality of clinical diagnosis and drug prescription for each disease. Learning curves were constructed to examine whether the quality improved with experience or after paraprofessionals interacted with veterinarians. Learning curves are used in clinical medicine to measure quality of service, and they are derived by graphically plotting performance against experience gained from the acquisition of new information or knowledge from prior experience [12]. Hopper et al. [13] argue that a steep learning curve implies that skills are acquired rapidly because the procedure is simple. In this particular case, a steep slope would mean service providers are consulting or learning from each other to build and maintain their reputation. Farmers’ belief updating curves were also constructed to examine whether farmers update their beliefs or change their beliefs about types of service providers. The slope of the curve measures the level of belief change or updating [10, 14]. Service providers were categorized into veterinarians and paraprofessionals. The latter were further differentiated by field and level of training. The mean scores in drug prescription for each category in each round were computed and plotted on a Cartesian axis in order to construct the learning curves. In addition, the total number of farmers seeking services from the different categories of service providers in each round was computed, and the results were used to construct farmers’ belief updating curves.

Non-parametric statistics were used to perform statistical tests because the Shapiro-Wilk test for normality and the Doornik-Hansen test for multivariate normality showed that the data violated the normality assumption. Since the normality assumption was violated, parametric tests were considered to be less powerful than the non-parametric tests because they do not assume normality [15]. A panel probit model with random-coefficient that allows for unobserved heterogeneity in farmers’ belief updating in each round was estimated to determine factors that influence farmers belief updating. In the model, belief updating is measured as a farmer’s decision to change to a different service provider from the previous service provider. A random effects model was chosen because (1) the observations are many, but the number of rounds are few (R = 4); thus a fixed effect model would give inconsistent estimates, and (2) a random effects model allows one to make inferences about the whole population, something that cannot be done with a fixed effects model [16]. Maddala [16] further notes that the probit model is well suited for estimating random effects because it produces correlations among errors yet logistic distribution is very restrictive for this purpose. Gibbons & Hedeker [17] used it to predict the probability of some doctors experiencing malpractice claims and in this paper it is used to determine factors that influence livestock farmers’ decision to change service provider.

Three models were estimated because of collinearity in the variables. In model one, sex of farmer, pay-offs of farmers, fees charged by service provider and livestock production systems were included in the model. Farmers’ education level and previous outcome were excluded because they were correlated with production system and pay-offs, respectively. In the model two, variable or production system was dropped, and farmers’ education was included. In model three, the pay-off variable was dropped, and the outcome variable was included and standardized beta values of the independent variables were reported because they show which of the independent variables have a greater effect on the probability to change service providers.

## Results

### Analysis of service quality by disease

Results from the Kruskal-Wallis and Kolmogorov-Smirnov non-parametric test showed that there is no statistical evidence to suggest that the scores of government animal health assistants in the pastoral areas in clinical diagnosis and prescription were different from the scores of the veterinarians (p < 0.05). The mean score achieved by government animal health assistants for identifying all signs of the disease (clinical diagnosis) were 58%, and the mean score for drug prescription was 98%. Veterinarians had a score of 53% for clinical diagnosis and 99% in drug prescription. Consequently, the term “veterinarian” as used in this paper includes both the veterinarians and the government animal health assistants trained in veterinary science, who acted as veterinarians in the role play in the pastoral area. Paraprofessionals include service providers with a diploma and or a certificate in agriculture or social science. Community animal health workers (CAHWs) are those service providers who have received some training in animal health services, but do not hold a diploma or certificate.

**Figure 1:**
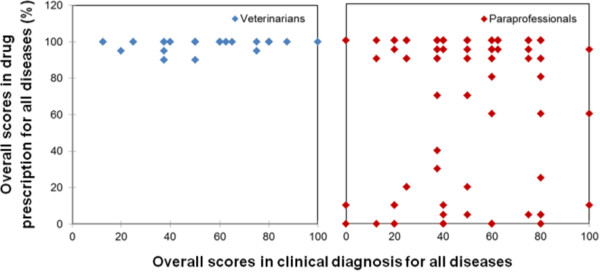
**The scatter diagram for veterinarians and paraprofessionals overall scores in diagnosis and prescription.**

Figure 1 is a scatter diagram of the overall scores in clinical diagnosis and drug prescription. Results show that the veterinarians’ average score in drug prescription was always close to 100%, but in identifying the signs of the respective diseases, sometimes the veterinarians scored below 50%. The results from Kolmogorov-Smirnov two-sample test showed that there was a statistically significant difference between paraprofessionals and veterinarians in drug prescription, but not in clinical diagnosis. Consequently, the subsequent discussion of the results will mainly focus on drug prescription as a measure of the quality of service.

**Figure 2:**
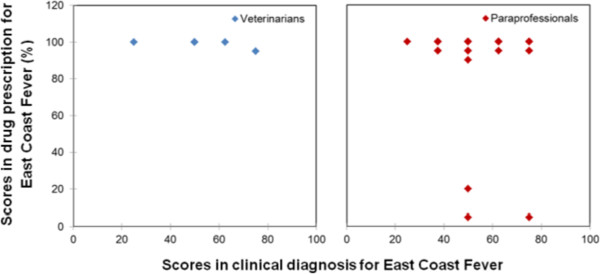
**Veterinarians and paraprofessionals scores in the treatment of East Coast Fever.**

**Figure 3:**
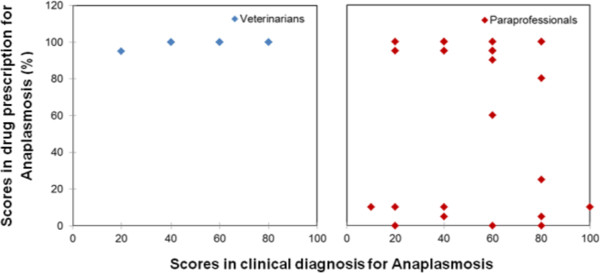
**Veterinarian and paraprofessional scores in the treatment of Anaplasmosis.**

**Figure 4:**
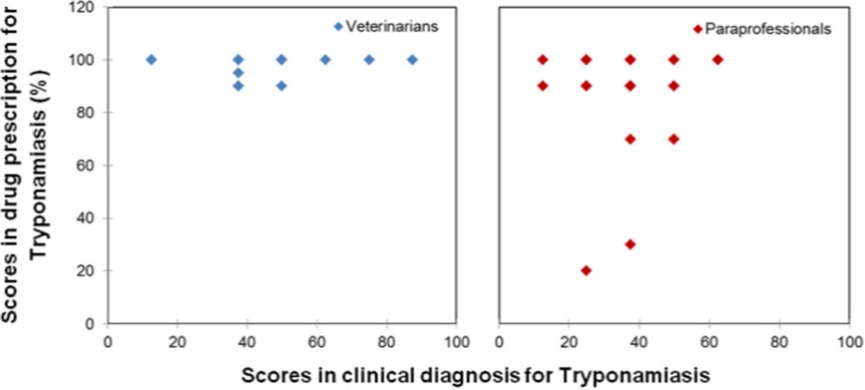
**Veterinarian and paraprofessional scores in the treatment of Tryponamiasis.**

**Figure 5:**
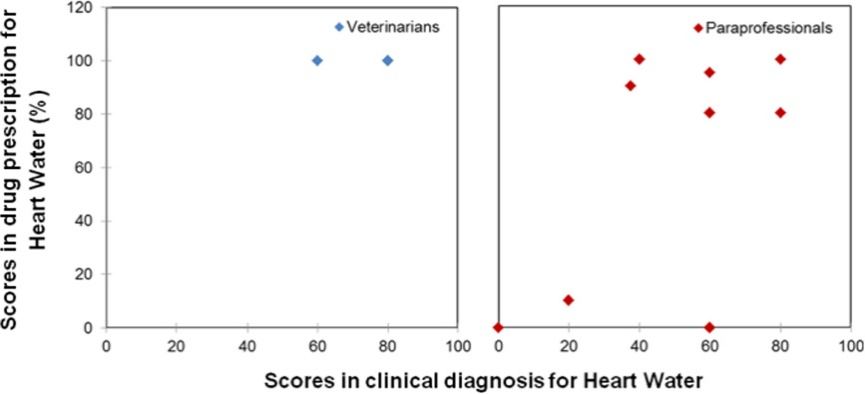
**Veterinarian and paraprofessional scores in the treatment of Heart Water.**

**Figure 6:**
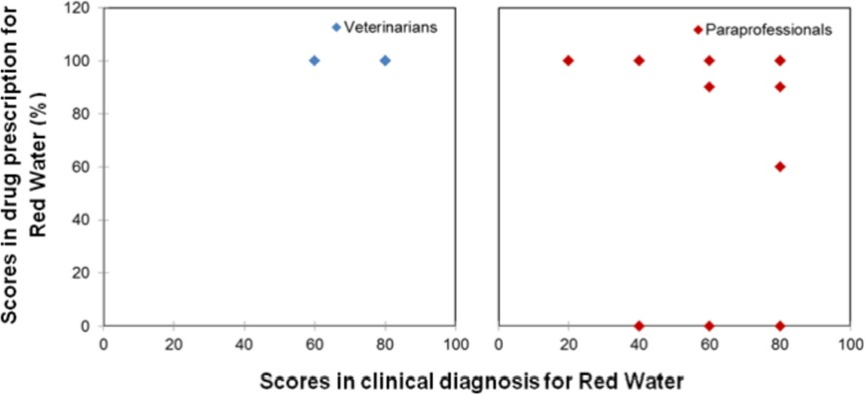
**Veterinarian and paraprofessional scores in the treatment of Red Water.**

The results of Kruskal-Wallis test revealed the presence of significant evidence to suggest that the level of drug prescription varied according to disease (p < 0.05). Figures 2, 3, 4, 5 and 6 are scatter diagrams for clinical diagnosis and prescription for each endemic disease. Six of the cases in ECF had a score of below 80% in drug prescription, four of which are from the intensive production system and two from the pastoral system. Three cases of the wrong prescription were from the same service provider, who had a diploma in crop science. This service provider was not interested in consulting with other service providers even after receiving the information that his prescription was inaccurate. He kept on prescribing Oxytetracycline, multivitamins and Imisol for ECF. The remaining case in the intensive system was handled by the service provider with a certificate in general agriculture. In the pastoral area, the two cases of inaccurate prescription were from a service provider who did not have any formal education, and the cases were recorded in rounds one and two. He also did not consult with veterinarians or other service providers. The prescription in both cases was only Oxytetracycline.

For Anaplasmoisis, there were thirteen cases with a score of below 80% in drug prescription; six cases were from the intensive system and seven from the pastoral system. In the intensive system, these service providers prescribed mainly multivitamins, Oxytetracycline, Butarex, Suriname, and Diminazene. In the pastoral areas, the prescriptions were mainly Oxytetracycline, multivitamins, and the following treatment: mixing either one litre or one-half litre of the cooking oil with one sachet OMO washing detergent. This sounds strange, but both service providers and farmers argued that using cooking oil and washing detergent yields positive results for Anaplasmosis.

In the case of Tryponamiasis, there were five cases where the score was below 80% in drug prescription, and all of these cases were from pastoral system. Two of the cases were from one service provider trained in social science, and three from service providers with no formal education. The drugs prescribed were Berenil and Oxytetracycline. Red Water and Heart Water had three cases each that recorded a score below 80% in drug prescription. All the cases were attributed to paraprofessionals without formal education, and the prescription for all the diseases in six cases was pen-strep.

### Learning curves and quality of veterinary services

The results from Kolmogorov-Smirnov test show that there is a statistically significant difference between the scores of paraprofessional and veterinarians regarding drug prescription, but not regarding clinical diagnosis (p < 0.05). The mean scores attained by paraprofessionals in clinical diagnosis and drug prescription were 50% and 72%, respectively. Veterinarians had a mean score of 56% in clinical diagnosis and 99% in drug prescription. Since scores of clinical diagnosis were not significantly different between paraprofessionals and veterinarians, accuracy in drug prescription was considered as a measure of service quality. The results from Kruskal-Wallis test show that the mean scores of drug prescription differed significantly by field of training and production system (p < 0.01). Also, only scores of drug prescription in rounds one and four were significantly different (p < 0.05).

**Figure 7:**
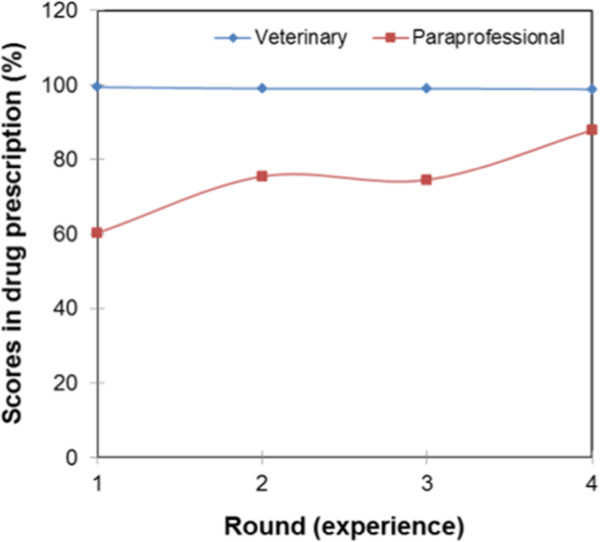
**Learning curves by type of the service provider.**

**Figure 8:**
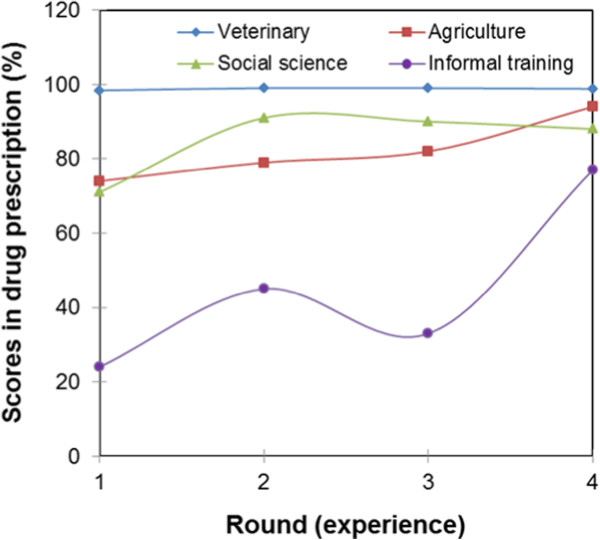
**Learning curves by field of training of service providers.**

**Figure 9:**
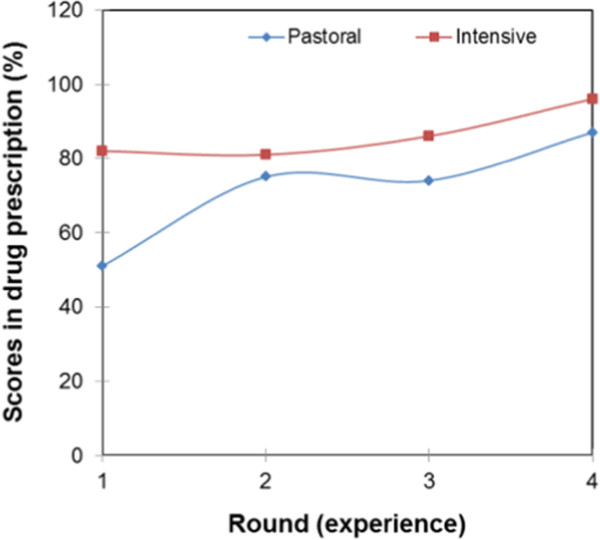
**Learning curves by livestock production system.**

Figure 7 shows the learning curves of paraprofessionals and veterinarians. The veterinarians’ curve shows that veterinarians are operating at maximum with an average score of 99% in drug diagnosis. The average score of paraprofessionals was below the 80% pass mark. In round one, the average score of paraprofessionals in drug prescription was 60%, and in round two it was 75% and no significant change was registered in round three. In round four, the average score increased to 88%. Figure 8 shows learning curves of service providers by field of training. The learning curves for service providers trained in veterinary science shows that they operate at maximum as expected. The average scores of service providers with a social science background were 71%, 91%, 90% and 88% in rounds one to four, respectively. The learning curve took the shape of an asymptotic curve as shown in Figure 8. Paraprofessionals trained in general agricultural had a slow, but gradually increasing learning curve with scores of 74%, 79%, 82%, and 94% in rounds one to four, respectively. The scores of paraprofessionals trained in agriculture and social science were not significantly different at p < 0.05. The learning curves of paraprofessionals with no formal education took the shape of a sigmoid curve, and the average score in drug prescription of non-educated paraprofessionals failed to reach 80% even with continued interaction between paraprofessionals and veterinarians. The score were 24%, 45%, 33% and 77% in rounds one to four, respectively. The learning curves in Figure 9 show that the quality of clinical veterinary services in the pastoral system is lower than that in the intensive system. The learning curve of intensive system is gradually increasing, while that of the pastoral system takes a sigmoidal shape.

### Demand for clinical services

**Figure 10:**
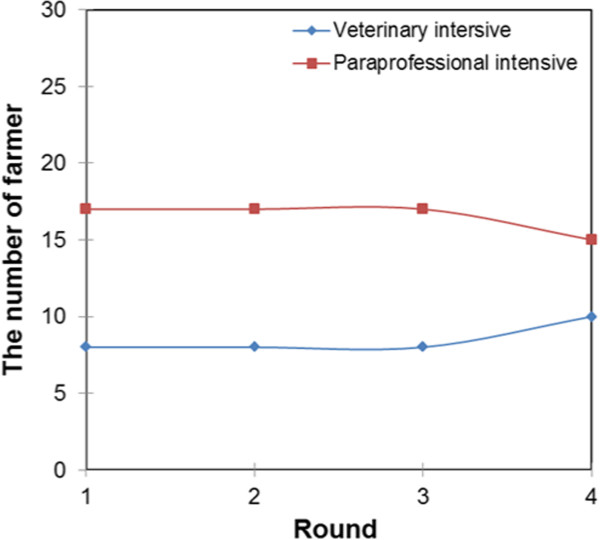
**Farmers’ belief updating curves about service providers in the intensive production system.**

**Figure 11:**
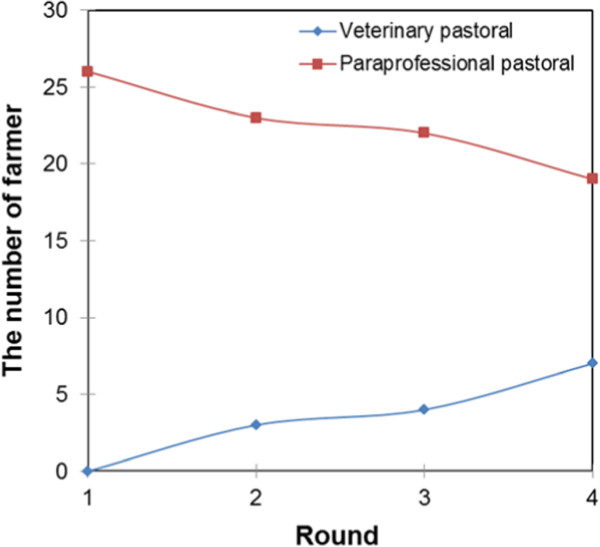
**Farmers’ belief updating curves about service providers in the pastoral system.**

Figures 10 and 11 present the farmers’ belief updating curves with regard to the services of veterinarians and paraprofessionals in the intensive and pastoral system, respectively. The curves show that, in the intensive system, farmers do not easily update their beliefs about paraprofessionals. The belief updating curves were perfectly inelastic even with experience of interaction up to round three. This means that even when farmers get negative results, they still go back to the same paraprofessional or seek services of another paraprofessional but not services of veterinarians. In round four, the demand for veterinary paraprofessional services declined while that of veterinarians increased. In the pastoral systems, the demand for the services of veterinarians gradually increased while that of paraprofessionals gradually decreased.

**Figure 12:**
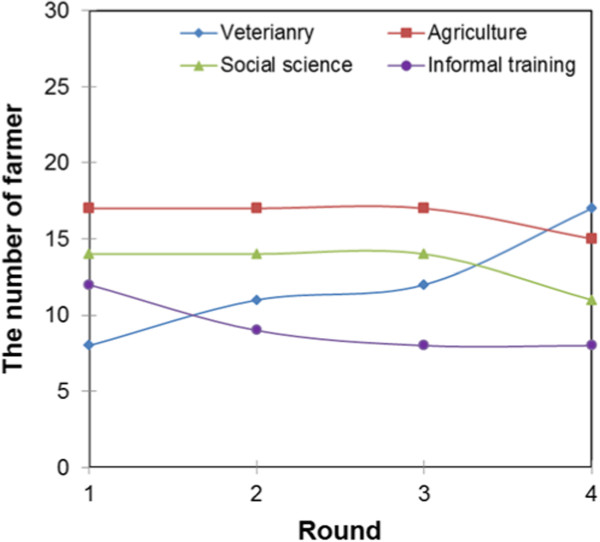
**Farmers’ belief updating curves about service providers of different fields of training.**

Figure 12 shows that service providers with agricultural training were found only in the intensive livestock system while those with no formal training and social science training were found only in the pastoral livestock production system. The farmers’ belief updating curves for service providers trained in social sciences and agriculture were inelastic between rounds one and three, and a decline was recorded in round four. The decline in round four can be associated with the increase in the demand for services of veterinary-trained service providers since the demand for service providers without formal education remain constant. The farmers’ belief updating curve for veterinary-trained service providers gradually increased while that of service providers without formal education gradually declined.

**Figure 13:**
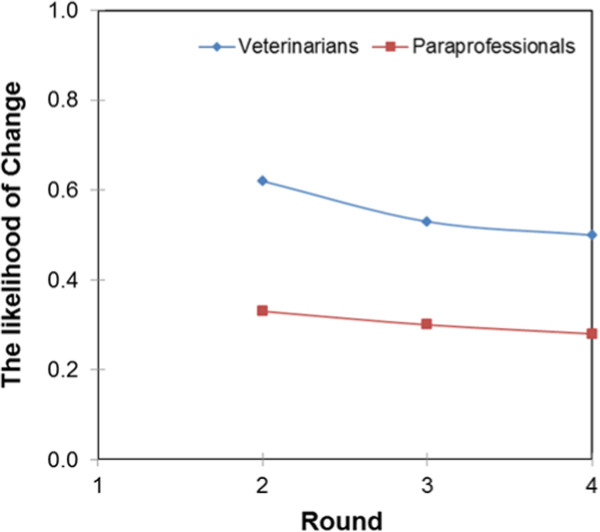
**The likelihood of changing to veterinarian or paraprofessional.**

As shown in Table 2, results from model one show that being female and having a high pay-off reduces the likelihood of changing service providers. Farmers in the intensive production system are more likely to change service providers than those in the pastoral system. As the descriptive statistics show, most of the changes were made from one paraprofessional to another and not to a veterinarian at least up to round four, see Figure 10 above. In model one; the livestock production system had a high significant effect on the decision to change, followed by pay-off and gender. In Model 2, results show that an educated farmer is more likely to change service providers than an uneducated farmer and education had a higher significant effect than pay-off. In Model 3, results revealed that the outcome of the previous transaction influences a farmer’s decision to change service providers, but the livestock production system had a higher significant effect, followed by outcome and sex. In all models, a fee charged by service providers in the previous transaction does not influence the decision to change providers. The likelihood that farmers change service providers was predicted using Stata post-estimation commands and the results showed that farmers are more likely to change to veterinarians than to paraprofessionals, as shown in Figure 13.

**Table 2:** **Random-effects panel probit model results for farmer’s decision to change a service provider**

	Probit model					
Independent variables	Model 1		Model 2		Model 3	
Female farmers	-0.530*	(-2.10)	-0.25	(-1.08)	-0.494*	(-1.97)
Farmers’ pay-off from previous transaction	-0.908***	(-3.42)	-0.465*	(-2.02)		
Fees charged in previous transaction	-0.117	(-0.47)	-0.01	(-0.04)	0.176	(-0.71)
Intensive livestock production systems	1.372***	(-4.75)			0.966***	(-3.73)
Farmers with education			0.581*	(-2.53)		
Previous outcomes					0.676**	(-2.76)
N	142		142		139	
Wald chi2(4)	25.68		11.31		20.8	

Standardized beta coefficients; t statistics in parentheses * p < 0.05, ** p < 0.01, *** p < 0.001.

## Discussion

The objective of this study was to determine whether quality of clinical services for cattle differ between veterinary paraprofessionals, examine farmer’s choices of veterinary advice and implications of these choices on the quality of clinical services. The results show that paraprofessionals trained in veterinary science are capable of handling endemic cattle diseases just like trained veterinary professionals. However, the ability of paraprofessionals who are not trained in veterinary science is significantly lower than those of the service providers trained in veterinary science. The main problem in drug prescription by paraprofessionals was in the treatment of ECF and Anaplasmoisis. The learning curve for crop trained service providers was slowly increasing while those of the social science trained service providers assumed an asymptotic curve. The learning curve for service providers with no formal education took a sigmoid shape. Hopper et al. [13] suggest that the slow rise learning is an indication of a difficult task, while the asymptotic curve can be associated with quick learning. However, in this particular case, the slow rise in the learning curve may be attributed to low propensity to consult, while asymptotic curve can be attributed to the high propensity to consult. The learning curves also reveal that paraprofessionals were not performing well from round one to three and the best performance was recorded in round four.

The poor performance in round one to three can be explained by the unwillingness of paraprofessionals to consult with veterinarians while improved performance in round four can be explained by the loss of farmers to other providers. As the feedback meeting held after the game revealed, there are trade-off in consulting with other veterinarians. On one hand, consulting with veterinarians may increase the likelihood of losing a client to a veterinarian because farmers would lose confidence in them and veterinarians would use that as an opportunity to discredit them in front of their clients. On the other hand, consulting with veterinarians helps paraprofessionals to save face in front of their clients by avoiding negative outcomes. Secondly, farmers take a long to change their beliefs about the paraprofessionals. Thus, paraprofessionals have limited incentives to consult with veterinarians to save face in front of the farmers. As revealed by farmers during the feedback meeting, they would go back to the same paraprofessionals even when the previous outcome was negative because they knew them and would always want to give them the benefit of the doubt. The third reason could be a language problem which limits non-educated paraprofessionals to interact with veterinarians since most of the veterinarians especially is pastoral areas are not from same ethnic background [9].

The results generally contradict the findings by IDL [18], Oakeley et al. [19] and Admassu et al. [20] which showed that informal trained paraprofessionals as capable of performing diagnosis and drug prescription. For example, Oakeley et al. [19] conducted a random survey of veterinary service providers, including Community Animal Health Workers (CAHWs), who were only trained on the job, to examine the level of accuracy in drug diagnoses among different types of service providers. Their results showed that 85% of the diagnoses made by CAHWs were accurate. However, Curran & MacLehose [21] dismissed this finding on grounds that they do not have proper research design to assess the level of drug prescription, and in any case no scores were presented. In addition, the three studies cited above do not consider the role of information, behaviour of farmers and service providers in making animal health management decisions in real life. Chilonda & Van Huylenbroeck [22] argue that the study of the behaviour and decision-making processes of farmers, service providers and their interactions in different livestock production systems is a key to development of sustainable policy options for successful delivery of quality veterinary services to small-scale farmers.

The role play game has been identified and tested in the literature as a tool that can serve as an accurate and consistent method of assessment and decision forecasting [23, 24]. The tool captures complexities about actors (farmers and service providers) decisions and behaviours without losing reality [25–27]. The results show that while paraprofessionals with no veterinary training were found to be of low quality compared with service providers with veterinary training, farmers changed their beliefs about non veterinary trained paraprofessionals rather slowly, thus providing few incentives for these paraprofessionals to provide quality services. The slow pace by which farmers were updating their beliefs about non veterinary trained service providers was because these paraprofessionals are known to farmers while veterinarians were not known to farmers. Improving relations between paraprofessionals and veterinarians, therefore, is a key to improving quality of veterinary services [9]. However, this depends on farmers’ ability to “punish” service providers who provide poor quality services by shifting to quality service providers. Model results show this depends on their education level, outcomes of the service, and gender.

The fee charged for the previous transaction was found not to have a significant impact on farmers’ decision to change their service provider. These findings are consistent with findings by Ahuja, et al. [28]. They found out that the price is not an important determinant of farmers’ decision to use services of an alternative service provider. In fact, Leonard [29] argues that the issue is not that farmers are poor and unable to afford veterinary services, but rather that farmers have failed to distinguish qualifications of different services providers and the quality of services they offer. The use of animal health cards or animal medical cards has a strong potential as a tool to enable farmers to distinguish and measure quality of clinical veterinary services. Farmers can use exercise books and service providers could be asked to write their diagnosis and prescription in these books.

The role play experiment used in this study assumes that farmers do not self-treat, yet in reality farmers do treat the animal themselves. Self-treatment as an option was excluded because to include it the game would mean promoting unethical behavior. Secondly, the game assumed that the risk of an animal dying even when treated correctly is zero, and yet an animal can actually die even with the right treatment because of delayed reporting and drug administration [30]. Thirdly, the experiment assumed that veterinarians are always available which is not always the case especially in the pastoral system. Lastly, the limited number of participants could limit validity of the results, but since “real” participants (farmers and service providers) were involved in the game, the results are still meaningful and valid.

## Conclusions

In summary, this paper presents a systematic study on how the interactions of farmers, veterinarians and paraprofessionals influence the quality of clinical veterinary services in rural Uganda. Results reveal the quality of veterinary services provided by paraprofessionals with veterinary training are not significantly different from those of veterinarians. However, the quality of services offered by paraprofessionals without veterinary training is significantly lower than that of veterinary trained service providers, but would improve as they interact with trained service providers. Even though services offered by paraprofessionals without veterinary training would increase in quality from continued interaction with veterinarians, there are challenges of sustaining paraprofessional interaction with veterinarians. As the results show increased risks of losing clients, limited number (availability) of veterinarians and the slow pace by which farmers update their beliefs impede paraprofessional and veterinary interaction. From a policy perspective, investment in two years of training for veterinary paraprofessionals is a promising strategy for improving the quality of veterinary services since farmers are willing to pay for the private clinical veterinary services.

## Endnotes

^a^The Merck Veterinary manual for veterinary professionals http://www.merckmanuals.com/merckmanualvet-book.htm.

^b^OIE technical disease cards http://www.oie.int/animal-health-in-the-world/technical-disease-cards/.

## Authors’ information

JI is a Doctoral Fellow at the Food Security Centre, University of Hohenheim and his research interest is focussed on application of New Institutional Economics to analyse food security and service delivery challenges in developing countries. RB is the Chair of “Social and Institutional Change in Agricultural Development” at the University of Hohenheim, Germany and one of her research interest is on the question of how rural services, including veterinary services, can be delivered more effectively to rural poor.
